# CCL17 acts as a novel therapeutic target in pathological cardiac hypertrophy and heart failure

**DOI:** 10.1084/jem.20200418

**Published:** 2022-06-10

**Authors:** Yang Zhang, Yicong Ye, Xiaoqiang Tang, Hui Wang, Toshiko Tanaka, Ran Tian, Xufei Yang, Lun Wang, Ying Xiao, Xiaomin Hu, Ye Jin, Haiyu Pang, Tian Du, Honghong Liu, Lihong Sun, Shuo Xiao, Ruijia Dong, Luigi Ferrucci, Zhuang Tian, Shuyang Zhang

**Affiliations:** 1 Department of Cardiology, Peking Union Medical College Hospital, Chinese Academy of Medical Sciences and Peking Union Medical College, Beijing, China; 2 Key Laboratory of Birth Defects and Related Diseases of Women and Children of Ministry of Education, State Key Laboratory of Biotherapy, West China Second University Hospital, Sichuan University, Chengdu, Sichuan, China; 3 Longitudinal Studies Section, Translational Gerontology Branch, National Institute on Aging, National Institutes of Health, Baltimore, MD; 4 Department of Central Research Laboratory, Peking Union Medical College Hospital, Chinese Academy of Medical Sciences and Peking Union Medical College, Beijing, China; 5 State Key Laboratory of Medical Molecular Biology, Department of Biochemistry and Molecular Biology, Institute of Basic Medical Sciences, Chinese Academy of Medical Sciences and Peking Union Medical College, Beijing, China; 6 Thermo Fisher Scientific (China) Co., Ltd, Changning, Shanghai, China

## Abstract

Circulating proteomic signatures of age are closely associated with aging and age-related diseases; however, the utility of changes in secreted proteins in identifying therapeutic targets for diseases remains unclear. Serum proteomic profiling of an age-stratified healthy population and further community-based cohort together with heart failure patients study demonstrated that circulating C-C motif chemokine ligand 17 (CCL17) level increased with age and correlated with cardiac dysfunction. Subsequent animal experiments further revealed that *Ccll7*-KO significantly repressed aging and angiotensin II (Ang II)–induced cardiac hypertrophy and fibrosis, accompanied by the plasticity and differentiation of T cell subsets. Furthermore, the therapeutic administration of an anti-CCL17 neutralizing antibody inhibited Ang II–induced pathological cardiac remodeling. Our findings reveal that chemokine CCL17 is identifiable as a novel therapeutic target in age-related and Ang II–induced pathological cardiac hypertrophy and heart failure.

## Teaser

Zhang et al. identified chemokine CCL17 as a contributor to age-related heart failure in humans and mice via regulating plasticity and differentiation of T cells. Their findings suggested CCL17 as a novel therapeutic target in age-related heart failure.

## Graphical Abstract

**Figure:**
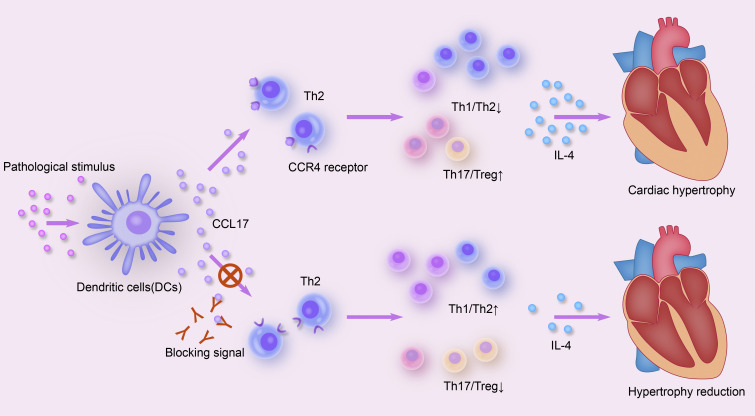

## Introduction

Pathological cardiac remodeling, characterized by left ventricular (LV) hypertrophy, cardiac fibrosis, and inflammation, is a determinant of the clinical course of heart failure (HF). Aging and the activation of the rennin-angiotensin system play an important role in pathological cardiac remodeling (Driver et al., 2008; Tang et al., 2017). Anti-aging treatments, such as caloric restriction and rennin-angiotensin system–inhibitor use, potentially improve HF by reducing cardiac inflammation. Evidence corroborating the potential benefit of inflammation suppression in reducing cardiovascular disease is increasing. For example, canakinumab, a therapeutic human monoclonal antibody targeting IL-1β, has been shown to significantly lower major adverse cardiovascular event rates (Ridker et al., 2017). However, the effectiveness of anti-inflammatory therapy in HF awaits full elucidation (Mann, 2015).

Chemokines and chemokine receptors are important components of the cytokines that orchestrate immune-cell migration and maintain homeostasis. Based on the features of amino-terminal cysteine residues, chemokines can be subdivided into four classes: CC, CXC, CX3C, and C. All chemokines perform their biological function via corresponding receptors (i.e., CCR, CXCR, CX3CR, and XCR molecules). Early research has established that C-C motif chemokine ligand 17 (CCL17) plays an important role in T cell development in the thymus, and it binds to C-C chemokine receptor 4 (CCR4) and displays chemotactic activity for T lymphocytes (predominantly T helper 2 [Th2]–subtype cells); thus, it is also typically referred to as the thymus and activation-regulated chemokine (TARC; Imai et al., 1996). Moreover, we (Ye et al., 2017; Ye et al., 2015; Ye et al., 2014) and other studies (Weber et al., 2011; Zernecke and Weber, 2014) have demonstrated that chemokine CCL17, an important regulator of atherosclerosis, is positively associated with coronary artery disease, independent of traditional cardiovascular risk factors. Chemokines are currently believed to be involved in all stages of cardiovascular response to injury and are considered a possible therapeutic target (Dusi et al., 2016; Mikolajczyk et al., 2021). However, the role of CCL17 in pathological cardiac hypertrophy is yet to be investigated.

Given the current lack of age-related molecules for HF treatment, the elucidation of underlying molecular mechanisms and discovery of potential targets through the combination of clinical cohorts and aging-disease models are warranted. Herein, we report CCL17’s tendency to display an age-dependent increase in population studies and potential role as a critical participant in age-related and angiotensin II (Ang II)–induced cardiac hypertrophy and HF. Our study developed a comprehensive perspective of the mechanism by which circulating CCL17 intrinsically bridges pathological cardiac dysfunction, thus confirming it as a potential novel therapeutic target.

## Results

### Circulating CCL17 expression exhibits an age-dependent increase and correlates with cardiac function status in patients with HF

**Figure 1.**
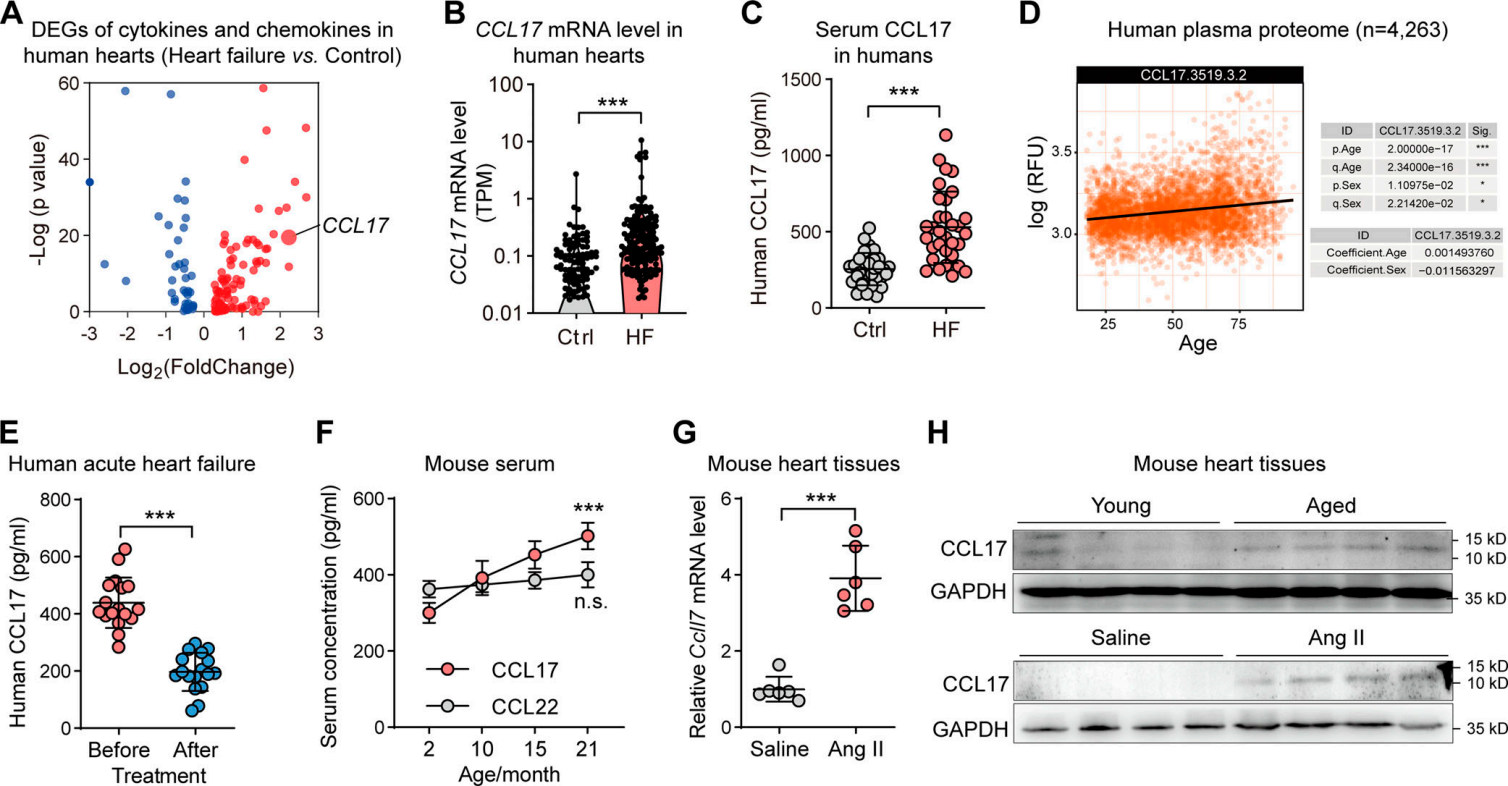
**Serum CCL17 exhibited an age-dependent increase and association with HF.** **(A)** The analysis of RNA sequencing in nonfailing donors and HF samples (*n* = 200 vs. 166) using the GEO database (GSE141910). **(B)** Levels of *CCL17* mRNA in nonfailing donors and HF samples (*n* = 200 vs. 166). **(C)** ELISA assay of serum CCL17 levels in normal controls and patients with HF (*n* = 32 biological replicates). **(D)** Levels of serum CCL17 from 4,263 young adults to nonagenarians (18–95 yr old) were analyzed using SOMAscan aptamer technology (https://twc-stanford.shinyapps.io/aging_plasma_proteome/). **(E)** Serum CCL17 levels before and after treatment of patients with acute decompensated HF (*n* = 17 biological replicates). **(F)** Expression of CCL17 and CCL22 in the serum of 2-, 10-, 15-, and 24-mo-old C57BL/6J background WT mice (*n* = 6 biological replicates). **(G)** qRT-PCR results showing the relative mRNA levels of *Ccl17* in 8–12-wk-old C57BL/6J background mice infused with saline or Ang II (*n* = 6 biological replicates). **(H)** CCL17 protein levels in Ang II–induced and age-related hypertrophic hearts. Western blotting showing the CCL17 protein levels in WT and *Ccl17*-KO mice at 4 mo (young) or 21 mo (aged) among C57BL/6J background mice (top). Western blotting showing the CCL17 protein levels of 8–12-wk-old C57BL/6J background WT and *Ccl17*-KO mice infused with saline or Ang II for 4 wk (bottom; *n* = 3 independent experiments). All data are presented as the mean ± SD. Statistical significance was tested using the unpaired (B–C and G) or paired Student’s *t*-test (E) and one-way ANOVA, followed by the Bonferroni post hoc test (F). ***, P < 0.001. TPM, transcripts per kilobase of exon model per million mapped reads; RFU, relative fluorescence unit.

To investigate the role of CCL17 in HF, we initially downloaded resources on LV transcriptomes from the Gene Expression Omnibus (GEO) database, which contained 366 samples, including 200 HF and 166 non-HF samples (GSE141910). We reanalyzed the RNA sequencing in nonfailing donors and HF samples. Compared with that in non-failing controls, CCL17 expression was significantly increased and ranked higher in patients with HF (Fig. 1, A and B). Similarly, our cohort found serum CCL17 levels to be significantly higher in patients with HF than in normal controls (Fig. 1 C).

**Figure S1.**
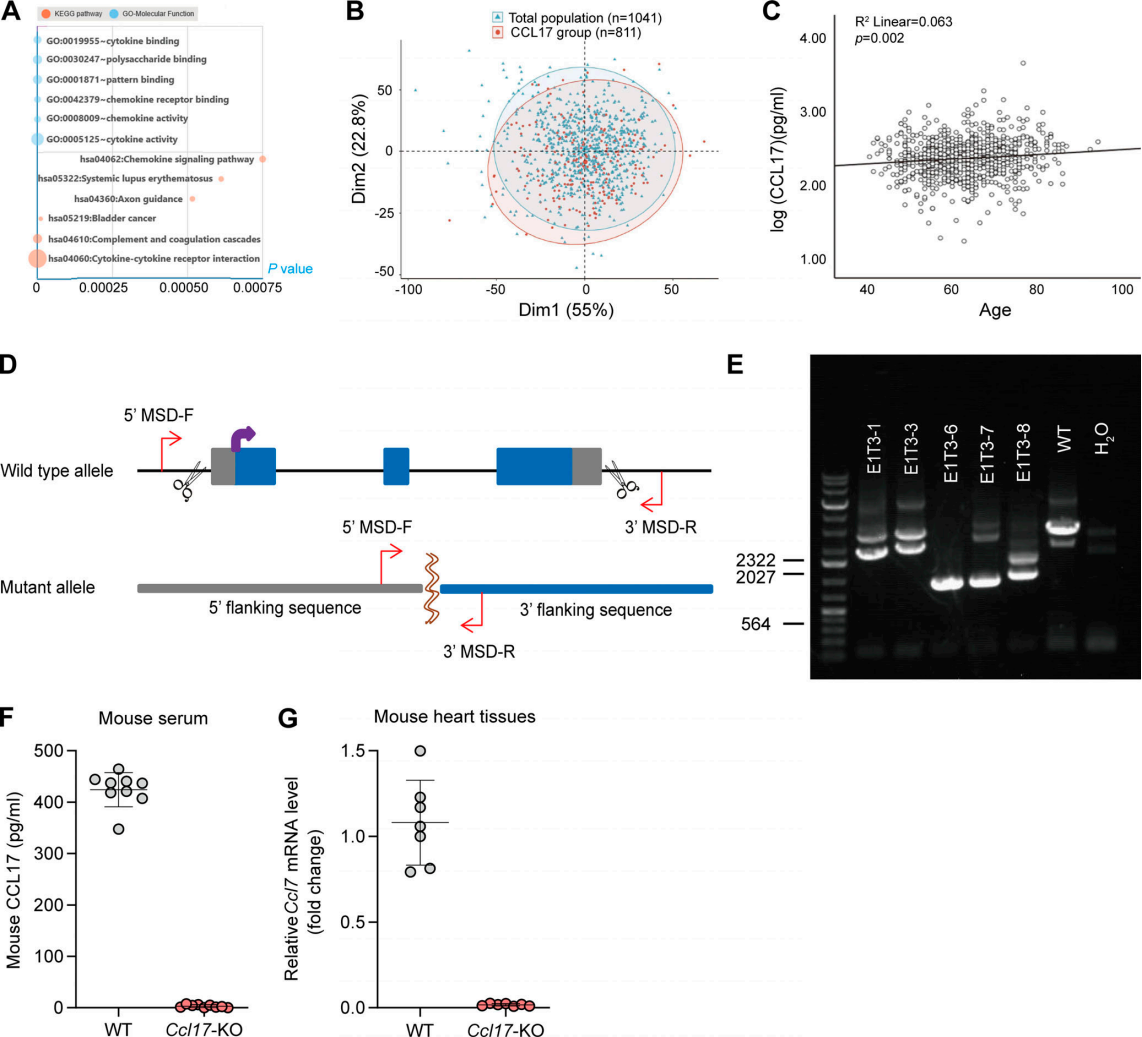
**Chemokine CCL17 exhibited an age-dependent increase and generation and validation of the *Ccl17*-KO mouse model.** **(A)** Top Kyoto Encyclopedia of Genes and Genomes (KEGG), Gene Ontology (GO)-Molecular Function, and GO-Biological Process terms enriched in age-associated SOMAmers. **(B)** PCA was used for the community-based Shunyi cohort study in China. We performed PCA for 13 clinical indexes in Table S3 (including CCL17 and BMI obtained from height and weight, so they were not repeated) of all patients, using function princomp in R and default parameters. The PCA results for individual patients were displayed using function fviz_pca_ind in the R package. **(C)** Age and serum CCL17 concentrations linear model in the Shunyi community population (*n* = 811). The linear regression model was adjusted for sex, BMI, hypertension, smoking status, LDL-C, and diabetes mellitus. P value = 0.002. **(D)** A schematic diagram depicting the construction of *Ccl17*-knockout mouse lines by using the CRISPR/Cas9 system. A couple of sgRNA target to the sequence of *Ccl17* was designed on upstream exon 1 and downstream exon 3 to form double-strand breaks. **(E)** PCR genotyping on genomic DNA obtained from C57BL/6J background WT (lane 6) and knockout (lane 3–5) mice (*n* = 3 independent experiments). We found that 1,704 bases were deleted in mouse #6, while 1,707 bases were deleted in mouse #7 by sequencing. **(F)** Quantitative analysis of CCL17 level in serum from 8–12-wk-old C57BL/6J background WT and *Ccl17*-KO mice (*n* = 9 biological replicates). **(G)** qRT-PCR results showing the relative mRNA levels of *Ccl17* in the heart from 8–12-wk-old WT and *Ccl17*-KO mice (*n* = 7 biological replicates). MSD, mutant sequence design.

The SOMAscan assay, a newly developed technology based on the slow off-rate modified aptamer (SOMAmer) that can capture and quantify numerous proteins in human bodily fluids, provides a promising alternative method for the establishment of links between circulating aging biomarkers and disease (Gold et al., 2012). Using SOMAscan aptamer technology, Lehallier et al. (2019) measured 2,925 plasma proteins from 4,263 young adults to nonagenarians (18–95 yr old) and developed a new bioinformatics approach that uncovered marked age-related, nonlinear alterations in the human plasma proteome. Through the public database they developed (https://twc-stanford.shinyapps.io/aging_plasma_proteome/), we found circulating CCL17 to be significantly associated with age (Fig. 1 D). Furthermore, we used this approach to conduct proteomic profiling of 240 healthy men and women within the 22–93-yr age range, and the subjects’ basic characteristics are described in Table S1 (Tanaka et al., 2018). A total of 1,301 SOMAers associated with chronological age were measured, among which 312, including CCL17, were shown to have differences among the oldest subjects in a basic model adjusted for sex, site, race, and batch (Table S2). To explore pathways that are most enriched in these proteins, a functional annotation clustering analysis was conducted using DAVID software. As shown in Fig. S1 A, inflammatory and cytokine pathways were the most enriched.

To confirm CCL17 results in proteomics analysis, an ongoing community-based cohort in the Shunyi district of China was used (Zhai et al., 2018). Circulating CCL17 levels were measured in 811 subjects (Table S3) and the CCL17 testing population was found to be matched to the total population by principal component analysis (PCA; Fig. S1 B). Using a linear regression model adjusted for confounding factors (sex, body mass index [BMI], hypertension, smoking status, low-density lipoprotein cholesterol [LDL-C], and diabetes mellitus), our results revealed that the serum CCL17 level was significantly associated with age (Fig. S1 C). Moreover, to explore the inextricable correlation between cardiac function status and circulating CCL17 levels, we collected clinical data and blood samples from 17 patients with acute decompensated HF before and after treatment (Table S5). Indeed, after receiving standard treatment for HF, the symptoms of all patients improved rapidly, as indirectly indicated by a decline in the level of amino-terminal pro-brain natriuretic peptide, a biomarker recommended by the 2021 European Society of Cardiology (ESC) guidelines for evaluating the severity and prognosis of HF (McDonagh et al., 2021). Concurrently, we found circulating CCL17 levels to decrease with cardiac function recovery (Fig. 1 E). In addition, further experiments demonstrated that CCL17 expression in mice serum increased with age. However, as another important CCR4-binding chemokine, CCL22 expression did not increase with age (Fig. 1 F).

Based on the above findings, we investigated the role of CCL17 in age-related and Ang II–induced cardiac dysfunction in the present study. We found CCL17 to be barely detectable; nonetheless, its expression remarkably increased at both mRNA and protein levels upon subjection to Ang II infusion, resembling that associated with aging (Fig. 1, G and H).

Collectively, the above findings suggest that CCL17 is a strong candidate target for aging and is potentially involved in the progression of HF.

### CCL17 deficiency blocks cardiac hypertrophy and fibrosis in aged mice

**Figure 2.**
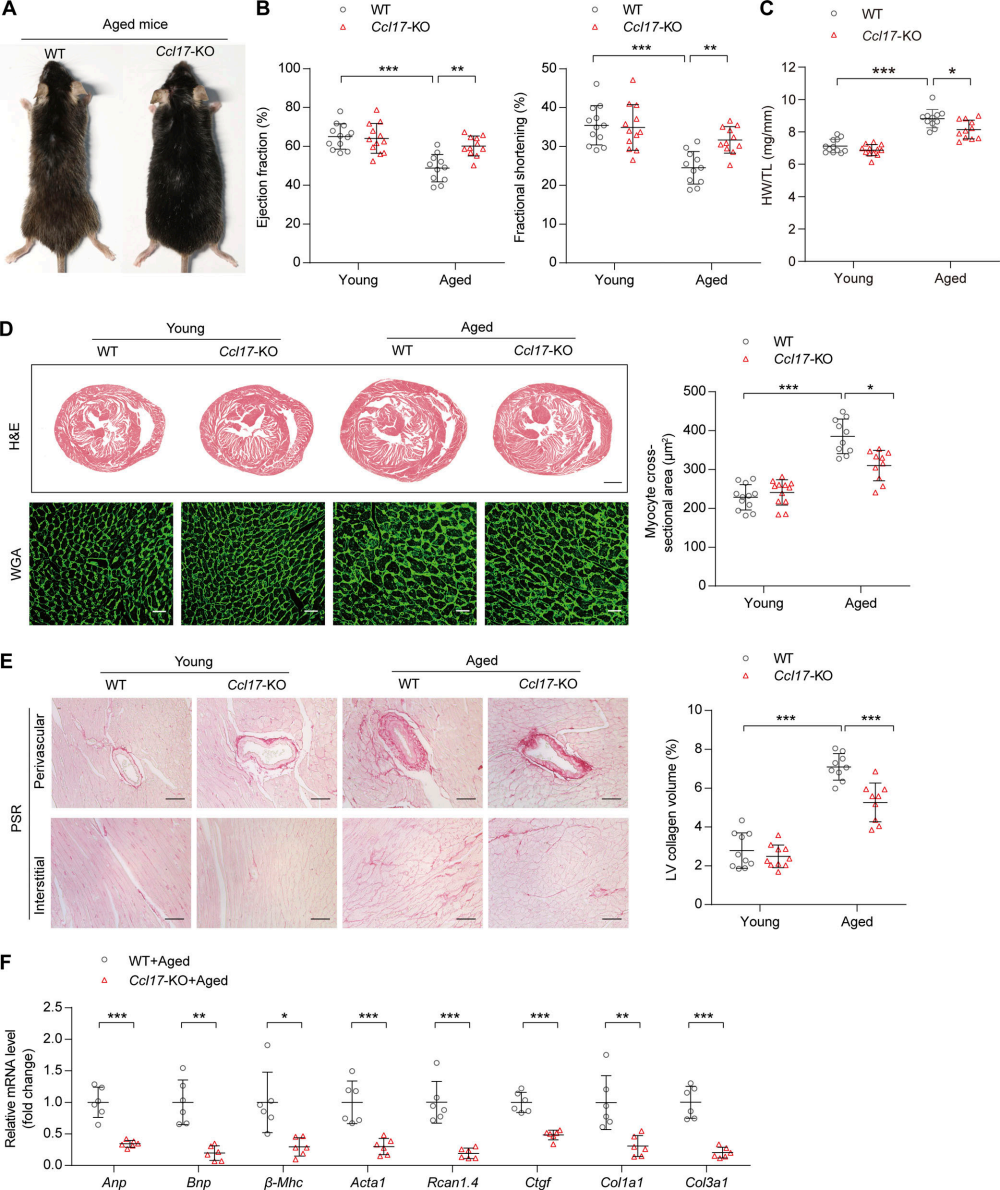
***Ccl17*-KO mice were protected from age-related cardiac hypertrophy and HF. (A)** Representative images of aged (21-mo-old) C57BL/6J background WT and *Ccl17*-KO mice. **(B)** Measurement of EF and FS in C57BL/6J background WT and *Ccl17*-KO mice at 4 mo (young) or 21 mo (aged; *n* = 11–12 biological replicates). **(C)** HW/TL ratios of C57BL/6J background WT and *Ccl17*-KO mice at 4 mo (young) or 21 mo (aged; *n* = 11–12 biological replicates). **(D)** Histological analysis of heart sections of C57BL/6J background WT and *Ccl17*-KO mice at 4 mo (young) or 21 mo (aged). Heart cross-sections were stained with H&E (top) to analyze hypertrophic growth of hearts, scale bars: 1 mm. WGA (bottom) staining of heart sections and corresponding group quantification data (right) to determine cell boundaries (*n* = 10–12 biological replicates). Scale bars: 30 µm. **(E)** PSR (left) staining and corresponding group quantification data (right) of C57BL/6J background WT and *Ccl17*-KO mice at 4 mo (young) or 21 mo (aged; *n* = 9–10 biological replicates). Scale bars: 50 µm. **(F)** qRT-PCR was performed to analyze the mRNA levels of cardiac function (*Anp*, *Bnp*), hypertrophy (*β-Mhc*, *Acta1*, *Rcan1.4*), and fibrosis (*Ctgf*, *Col1a1*, *Col3a1*) markers in aged (21-mo-old) C57BL/6J background WT and *Ccl17*-KO mice (*n* = 6 biological replicates). All data are presented as the mean ± SD. Statistical significance was tested using one-way ANOVA, followed by the Bonferroni post hoc test (B–E). The unpaired Student’s *t*-test was used for equal variance and the Welch *t*-test for unequal variance (F). *, P < 0.05; **, P < 0.01; ***, P < 0.001.

**Figure S2.**
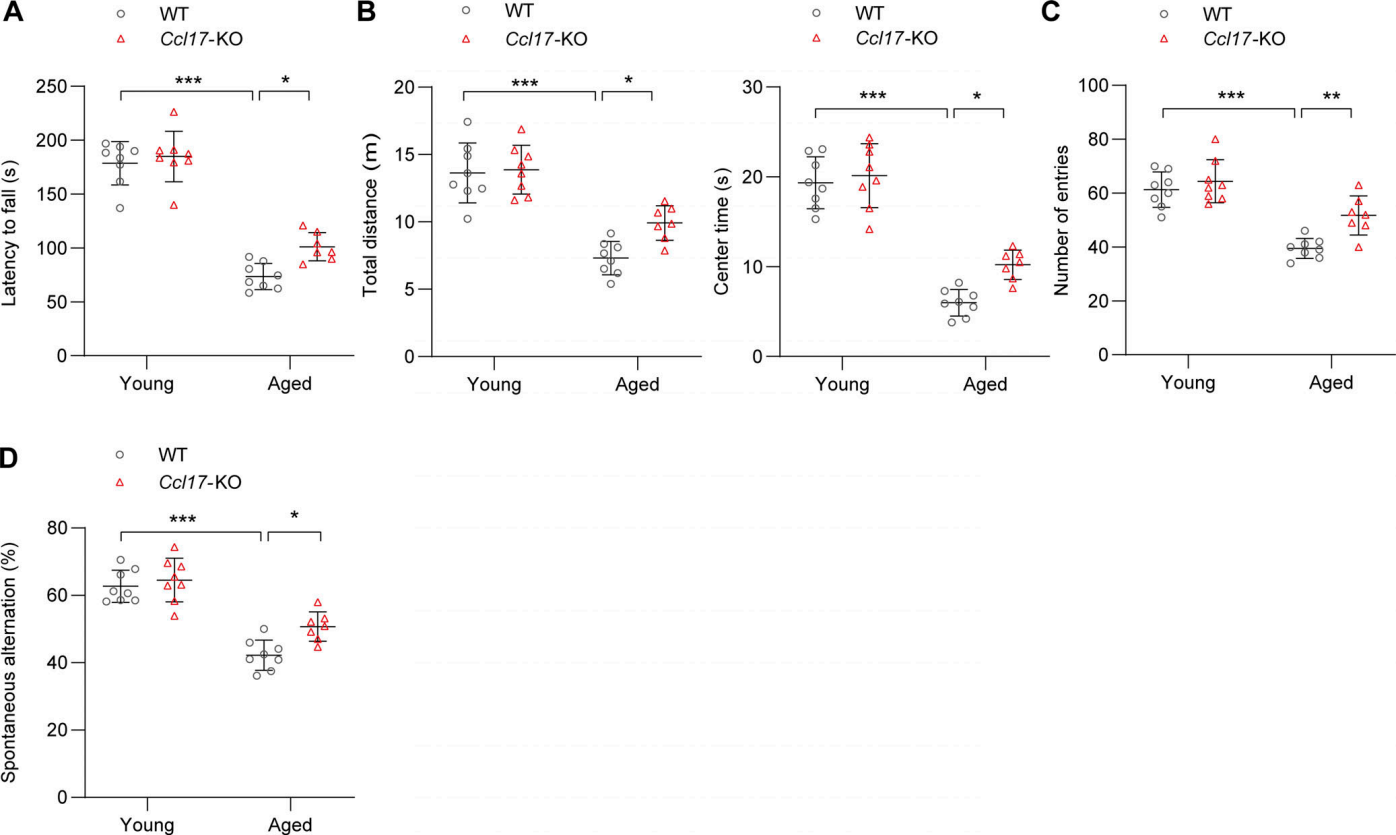
**Age-related changes in grip and behavior. (A)** Latency to fall off a rotating rod (s) in the rotarod test of C57BL/6J background WT and *Ccl17*-KO mice at 4 mo (young) or 21 mo (aged; *n* = 7–8 biological replicates). **(B)** Open-field test. Total distance traveled (m) and center time (s) for each 5-min block of testing of C57BL/6J background WT and *Ccl17*-KO mice at 4 mo (young) or 21 mo (aged; *n* = 7–8 biological replicates). **(C and D)** The number of arm entries (C) and spontaneous alternation score (D) were measured in the Y-maze spontaneous alternation test to evaluate the working spatial memory of C57BL/6J background WT and *Ccl17*-KO mice at 4 mo (young) or 21 mo (aged; *n* = 7–8 biological replicates). All data are presented as the mean ± SD. Statistical significance was tested using one-way ANOVA followed by Bonferroni post hoc test (A–D). *, P < 0.05; **, P < 0.01; ***, P < 0.001.

To investigate the role of increased circulatory CCL17 expression during age-related maladaptive hypertrophy and cardiac remodeling, global germ-line *Ccl17*-KO mice were grown using the CRISPR/Cas9 system in our laboratory (Fig. S1, D and E). When CCL17 was knocked out, it was found to be undetectable in serum (Fig. S1 F) and heart tissues (Fig. S1 G). Thereafter, the male *Ccl17*-KO mice and their control WT littermates were maintained for 21 mo. Remarkably, aged *Ccl17*-KO mice exhibited more youthful characteristics (such as hair) than WT littermates (Fig. 2 A). In addition, compared with their control WT littermates, aged *Ccl17*-KO mice exhibited increased motor function (Fig. S2 A) and locomotor activity (Fig. S2 B, left), reduced anxiety-like behavior (Fig. S2 B, right), and improved spatial working memory (Fig. S2, C and D). These results suggested that CCL17 deficiency potentially improves the overall health status of aged mice.

**Figure S3.**
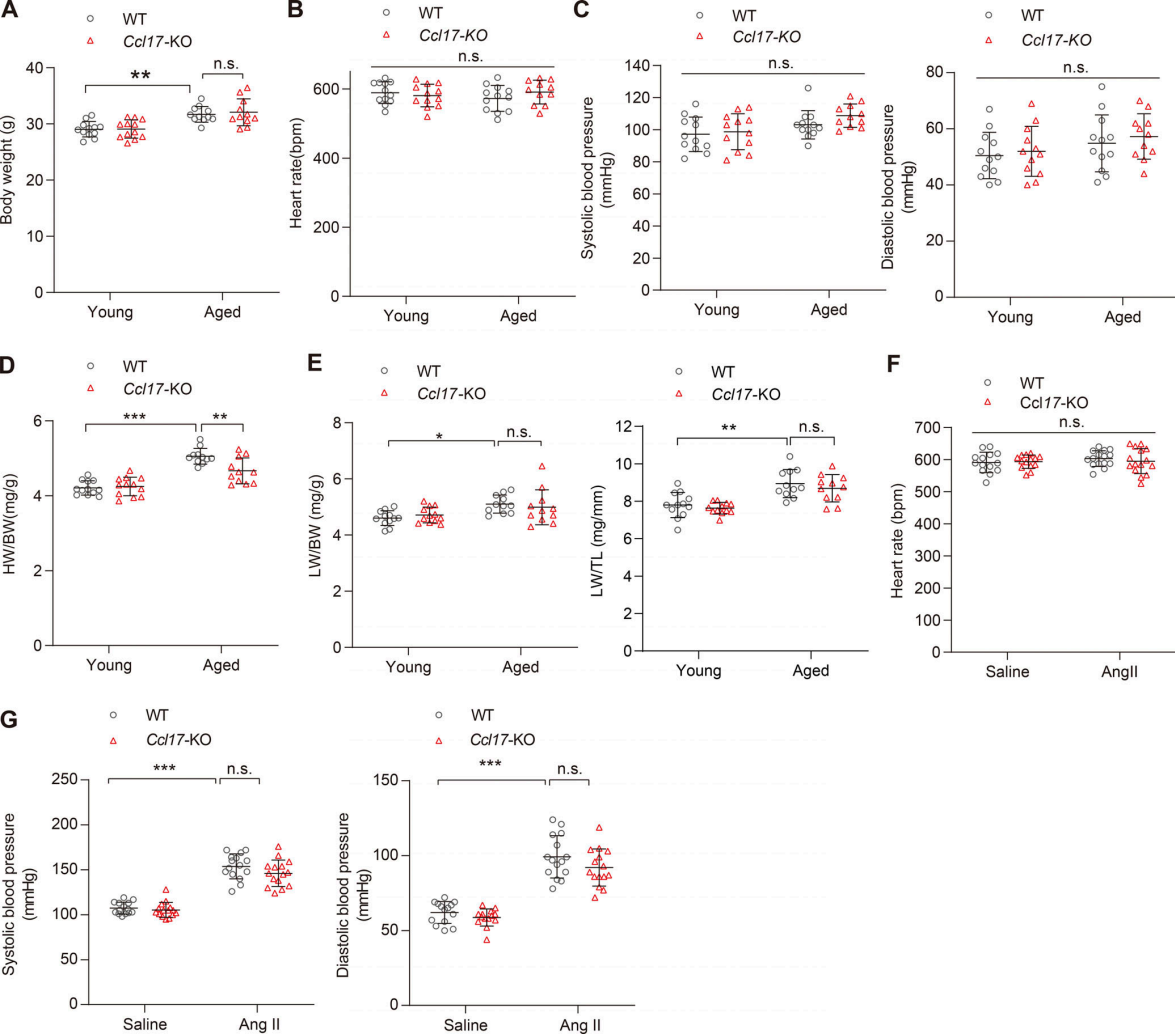
**Basic characteristics of WT and *Ccl17*-KO mice in age-related and Ang II–induced models. (A–C)** The body weight (A), heart rates (B), and blood pressures (C) of C57BL/6J background WT mice and *Ccl17*-KO mice at 4 mo (young) and 21 mo (aged; *n* = 11–12 biological replicates). **(D)** The ratios of HW/BW of young (4-mo-old) or aged (21-mo-old) C57BL/6J background WT mice and *Ccl17*-KO mice (*n* = 11–12 biological replicates). **(E)** The ratios of lung weight to body weight (LW/BW) or lung weight to TL (LW/TL) of C57BL/6J background WT and *Ccl17*-KO mice at 4 mo (young) or 21 mo (aged; *n* = 11 biological replicates). **(F)** Heart rates of 8–12-wk-old C57BL/6J background WT and *Ccl17*-KO mice infused with saline or Ang II for 4 wk (*n* = 14–15 biological replicates). **(G)** Systolic and diastolic blood pressures of 8–12-wk-old C57BL/6J background WT and *Ccl17*-KO mice infused with saline or Ang II for 4 wk (*n* = 14–15 biological replicates). All data are presented as the mean ± SD. Statistical significance was tested using one-way ANOVA followed by Bonferroni post hoc test (A, B, and D–G) or nonparametric Kruskal-Wallis test with post Dunn’s multiple comparisons test (C). *, P < 0.05; **, P < 0.01; ***, P < 0.001.

Subsequently, we further comprehensively evaluated the main cardiac indicators in aged mice. Echocardiography revealed that *Ccl17*-KO effectively improved cardiac systolic dysfunction (ejection fraction [EF] and fractional shortening [FS]) in aged mice (Fig. 2 B). No differences were observed in body weight (BW), heart rate, and blood pressure between aged WT and *Ccl17*-KO mice (Fig. S3, A–C). At baseline, aged mice had greater heart weight (HW)/tibia length (TL) and HW/BW ratios than young mice. Compared with those in the aged WT mice group, HW/TL and HW/BW ratios were decreased in the aged *Ccl17*-KO group (Fig. 2 C and Fig. S3 D), and no changes were detected in lung weight between the two aged-mice groups (Fig. S3 E). In addition, histological analysis was used to analyze age-related cardiac hypertrophy and fibrosis. H&E, wheat germ agglutinin (WGA), and picrosirius red (PSR) staining revealed increased heart size and fibrosis in the aged WT mice compared with that in the young WT mice. Remarkably, the pathological cardiac remodeling and fibrosis were attenuated in the aged *Ccl17*-KO mice (Fig. 2, D and E). Additionally, quantitative real-time PCR (qRT-PCR) was performed to analyze the mRNA levels of genes related to cardiac function (atrial natriuretic peptide [*Anp*] and brain natriuretic peptide [*Bnp*]), hypertrophy (myosin heavy chain β [*β-Mhc*], α-sarcomeric actin [*Acta1*], and regulator of calcineurin 1.4 [*Rcan1.4*]) and fibrosis (connective tissue growth factor [*Ctgf*], α-1 type I collagen [*Col1a1*], and collagen type III α-1 chain [*Col3a1*]) in aged mice, and we observed that the expression of all these genes was inhibited by CCL17 deficiency in aged mice (Fig. 2 F). These findings imply that CCL17 is involved in age-related cardiac hypertrophy and HF.

### CCL17 deficiency inhibits Ang II**–**induced cardiac hypertrophy and dysfunction

**Figure 3.**
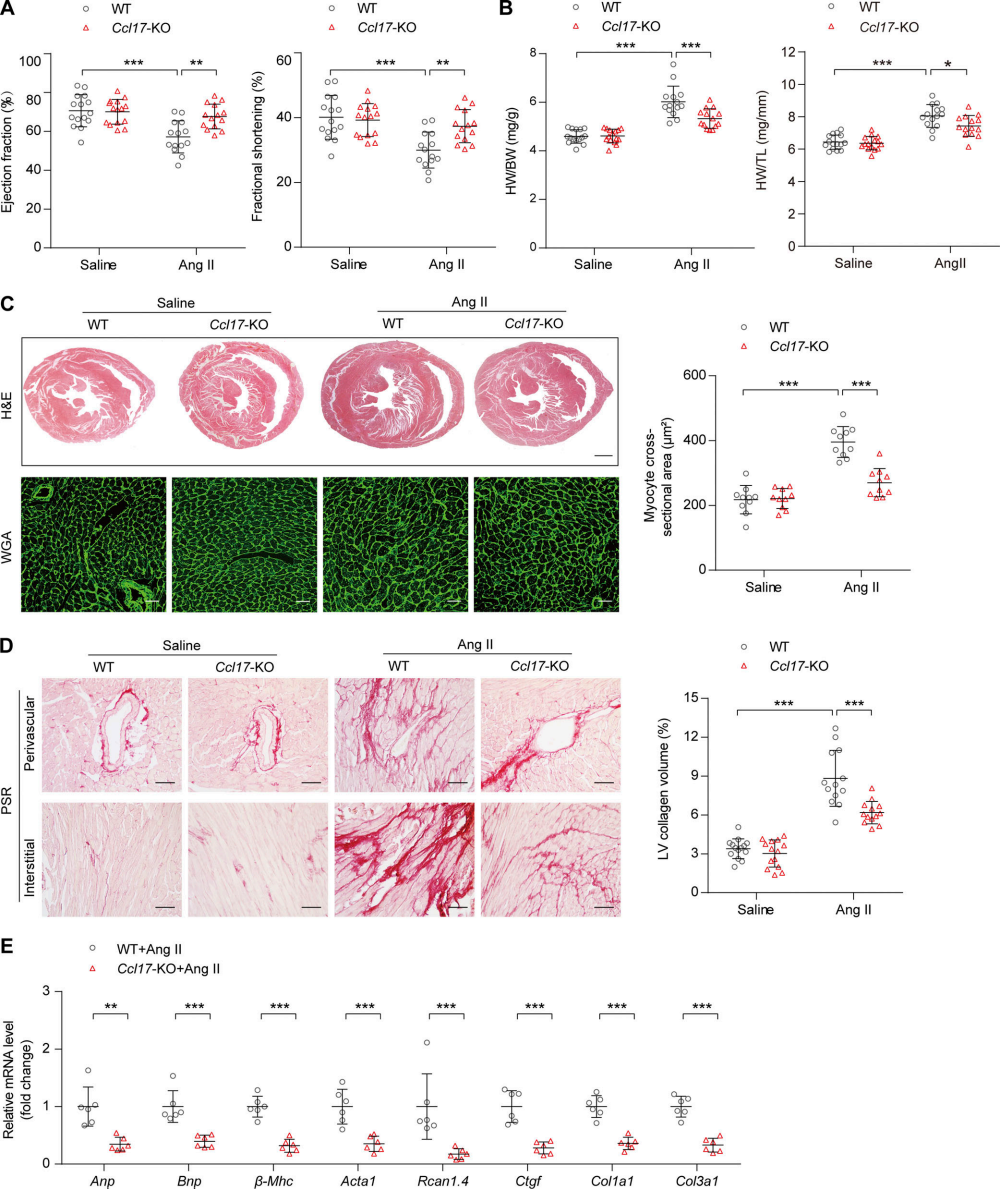
***Ccl17*-KO alleviated Ang II–induced cardiac hypertrophy and HF. (A)** EF and FS of 8–12-wk-old C57BL/6J background WT and *Ccl17*-KO mice treated with saline or Ang II for 4 wk (*n* = 14–15 biological replicates). **(B)** HW/BW and HW/TL ratios of 8–12-wk-old C57BL/6J background WT and *Ccl17*-KO mice treated with saline or Ang II (*n* = 14–15 biological replicates). **(C)** Histological analysis of heart sections from 8–12-wk-old C57BL/6J background WT and *Ccl17*-KO mice following saline or Ang II infusion. Heart cross-sections were stained with H&E (top) to analyze hypertrophic growth of hearts. Scale bars: 1 mm. WGA (bottom) staining of heart sections and corresponding group quantification data (right) to determine cell boundaries (*n* = 10 biological replicates). Scale bars: 30 µm. **(D)** PSR (left) staining and corresponding group quantification data (right) were performed to determine cardiac fibrosis of the hearts from 8–12-wk-old C57BL/6J background WT and *Ccl17*-KO mice treated with saline or Ang II (*n* = 13–14 biological replicates). Scale bars: 50 µm. **(E)** qRT-PCR was performed to analyze the mRNA levels of cardiac function (*Anp*, *Bnp*), hypertrophy (*β-Mhc*, *Acta1*, *Rcan1.4*), and fibrosis (*Ctgf*, *Col1a1*, *Col3a1*) markers in 8–12-wk-old C57BL/6J background WT and *Ccl17*-KO mice treated with Ang II (*n* = 6 biological replicates). All data are presented as the mean ± SD. Statistical significance was tested using one-way ANOVA, followed by the Bonferroni post hoc test (A–D). The Kolmogorov–Smirnov normality test was used to assess data distribution. If the data were not normally distributed, log transformation was used before analysis of variance (E). The unpaired Student’s *t*-test was used for equal variance and Welch *t*-test for unequal variance (E). *, P < 0.05; **, P < 0.01; ***, P < 0.001.

To explore the role of CCL17 in the heart, *Ccl17*-KO mice and WT littermates were treated with saline or Ang II, a key molecule in pathological cardiac hypertrophy that recapitulates the phenotype displayed in aged mice (Tang et al., 2017). After Ang II administration via an osmotic pump for 4 wk, blood pressure increased immediately; however, there was no impact on heart rate (Fig. S3, F and G). Compared with the control group, the *Ccl17*-KO group experienced a reversal of Ang II–induced cardiac dysfunction (decreases in EF and FS) and exhibited inhibitions of the increased HW/BW and HW/TL ratios (Fig. 3, A and B). Histological analysis with H&E and WGA staining revealed that the enlarged myocyte area induced by Ang II infusion was ameliorated in the *Ccl17*-KO mice (Fig. 3 C). In addition, the *Ccl17*-KO mice displayed less cardiac fibrosis than the WT mice (Fig. 3 D). Accordingly, the mRNA levels of genes related to cardiac function, hypertrophy, and fibrosis were also decreased (Fig. 3 E).

### *Ccl17* knockout ameliorates Ang II**–**induced cardiac inflammatory response

**Figure 4.**
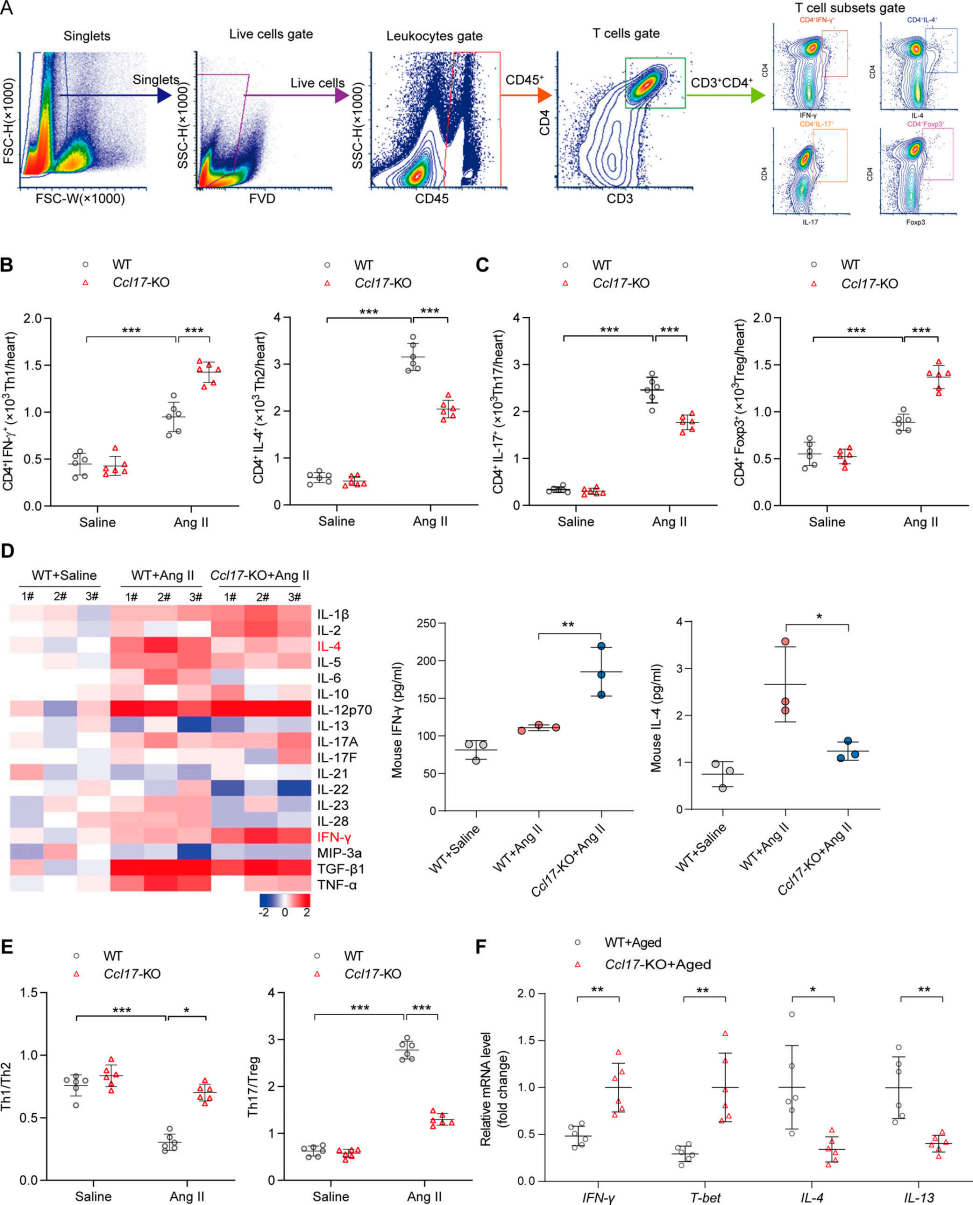
**CCL17 was involved in regulating T cell balance in Ang II–induced pathological cardiac hypertrophy and HF. (A)** Flow cytometry gating strategy to identify T cell distribution within the cardiac tissue. Cardiac mononuclear cells were initially gated on singlets, and live cells were selected. Thereafter, live cells were characterized by the expression of the CD45^+^ marker (leukocytes). Finally, among the leukocytes, CD3^+^CD4^+^ T cells were further analyzed for IL-4, IFN-γ, Foxp3, or IL-17 expression. **(B and C)** Flow cytometry analysis of T cell subsets in 8–12-wk-old C57BL/6J background WT and *Ccl17*-KO mice treated with saline or Ang II for 4 wk in heart tissues (*n* = 6 biological replicates). **(D)** Th1/Th2/Th17 cytokines heatmap showed dynamic changes in 8–12-wk-old C57BL/6J background WT and *Ccl17*-KO mice treated with saline or Ang II for 4 wk in heart tissues (left). Quantitative results of IFN-γ and IL-4 expression levels in heart tissues are shown (right; *n* = 3 biological replicates). **(E)** Th1/Th2 and Th17/Treg ratios from 8–12-wk-old C57BL/6J background WT and *Ccl17*-KO mice following saline or Ang II infusion for 4 wk (*n* = 6 biological replicates). **(F)** Relative mRNA levels of Th1/2 cytokine in C57BL/6J background WT and *Ccl17*-KO aged mice (21 mo old) heart tissues (*n* = 6 biological replicates). All data are presented as the mean ± SD. The Kolmogorov–Smirnov normality test was used to assess data distribution. Statistical significance was tested using one-way ANOVA, followed by the Bonferroni post hoc test (B–E). The unpaired Student’s *t*-test was used for equal variance and Welch *t*-test for unequal variance (F). *, P < 0.05; **, P < 0.01; ***, P < 0.001.

**Figure S4.**
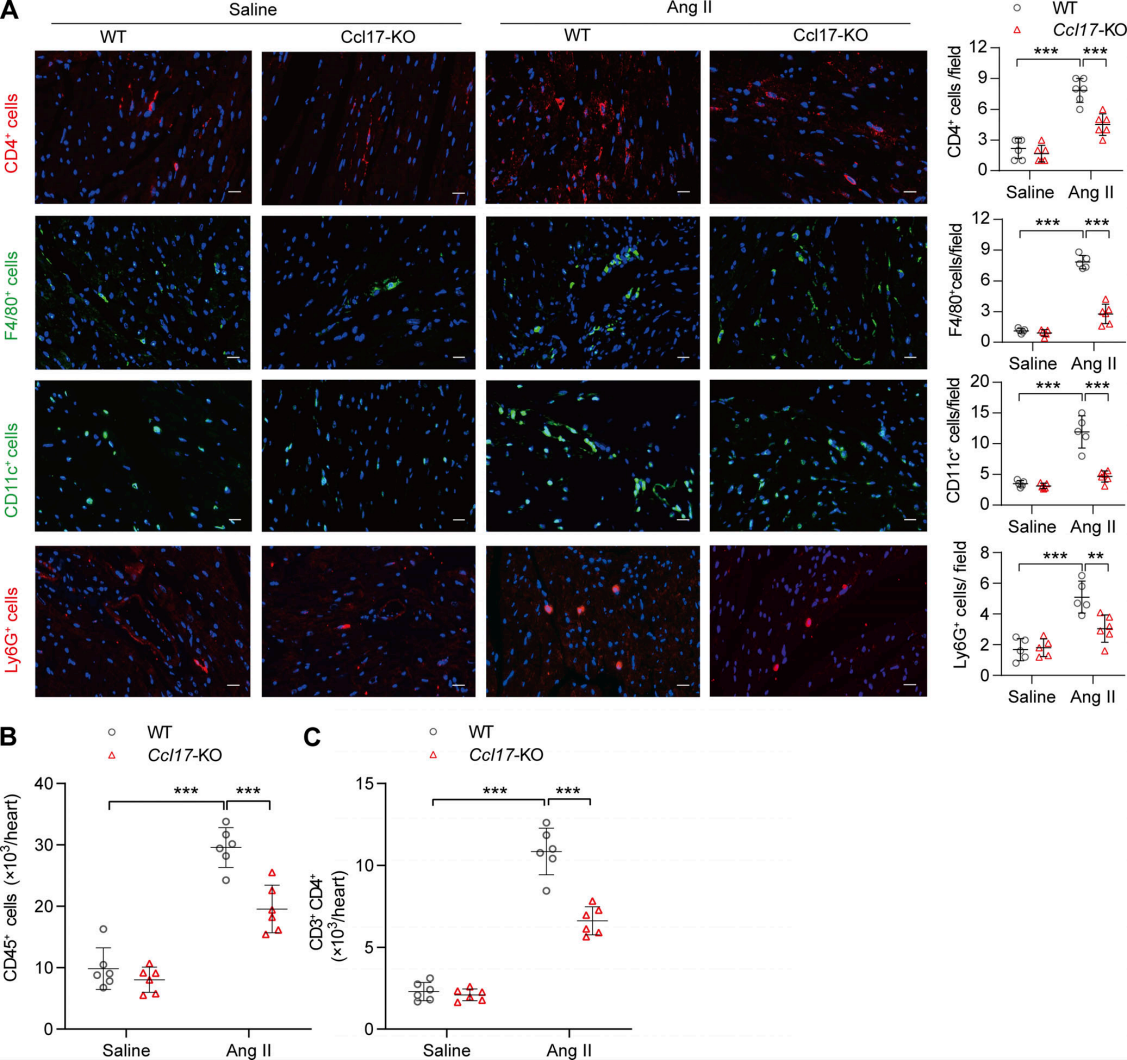
**Accumulation of CD45**^+^ **cells, CD4**^+^ **T cells, macrophages, DCs, and neutrophils within the cardiac tissue of WT mice after infusion with Ang II for 4 wk. (A)** Immunofluorescence analysis of CD4^+^ T cells (anti-CD4), macrophages (anti-F4/80), DCs (anti-CD11c), and neutrophils (anti-Ly6G) expression within the cardiac tissue of 8–12-wk-old C57BL/6J background WT mice after infused with Ang II for 4 wk (*n* = 6 biological replicates). Scale bars: 20 μm. **(B and C)** Flow cytometry analysis of CD45^+^ T cells (B) and CD3^+^CD4^+^ T cells (C) of 8–12-wk-old C57BL/6J background WT and *Ccl17*-KO mice treated with saline or Ang II for 4 wk in heart tissues (*n* = 6 biological replicates). All data are presented as the mean ± SD. Statistical significance was tested using one-way ANOVA followed by Bonferroni post hoc test (A–C). **, P < 0.01; ***, P < 0.001.

CCL17 is an important chemokine secreted by dendritic cells (DCs) involved in T cell activation (Alferink et al., 2003). Moreover, CD4^+^ T cells play a pivotal role in pathological cardiac remodeling, which predominantly induces cardiac hypertrophy by promoting myocardial fibrosis (Laroumanie et al., 2014). In this sense, we performed immunological staining and flow cytometry (see the gating strategy in Fig. 4 A) analysis to examine T cells in cardiac tissues. Our histological results found marked increases in the infiltration of CD4^+^ T cells, macrophages, DCs, and neutrophils in Ang II–induced hypertrophic hearts, while this effect was reduced in CCL17 deficient mice (Fig. S4 A). Accordingly, flow cytometry results indicated that there was a significant inflammatory response and marked infiltration of CD45^+^ (leukocytes; Fig. S4 B) and CD3^+^CD4^+^ T (Fig. S4 C) cells in hearts with Ang II–induced hypertrophy, and this phenomenon was repressed in the *Ccl17*-KO mice.

Of the Th subsets, cardiac Th1, Th2, Th17, and immunomodulatory regulatory T cell (Treg) populations all increased robustly following a 4-wk Ang II infusion, and CCL17 knockout was found to attenuate Th2 and Th17 response while relatively increased the expression of Th1 and Treg cells (Fig. 4, B and C). To further confirm the effect of CCL17 on T cell subsets, a protein microarray was used to measure the dynamic changes of cytokines produced by Th1/Th2/Th17 cells. As shown in Fig. 4 D, an overall inflammatory trend was observed in failing hearts. Compared with that in the WT group following Ang II infusion, Th2 cytokines (IL-4) were decreased, and Th1 cytokines (such as IFN-γ) exhibited a slight upward trend in *Ccl17*-KO mice. In addition, flow cytometry data from the failing hearts of *Ccl17*-KO mice demonstrated an increase in the Th1/Th2 cell ratio and a decrease in the Th17/Treg cell ratio compared with that of WT mice after infusion with Ang II (Fig. 4 E). Similarly, our results demonstrated that levels of Th1 cytokine IFN-γ and its activator, T-bet, were higher, while those of Th2 cytokines IL-4 and IL-13 were lower than those in the aged WT group after *Ccl17* knockout (Fig. 4 F). Therefore, the secretion of profibrotic factors, especially the Th2 cytokine IL-4 and Th17 cytokine IL-17, was reduced in *Ccl17*-KO mice, implying that CCL17 potentially contributes to cardiac remodeling through the T cell balance.

Taken together, these results suggest that CCL17 potentially participates in T cell polarization during pathological cardiac remodeling.

### Therapeutic administration of CCL17 neutralizing antibodies inhibits cardiac remodeling and dysfunction

**Figure 5.**
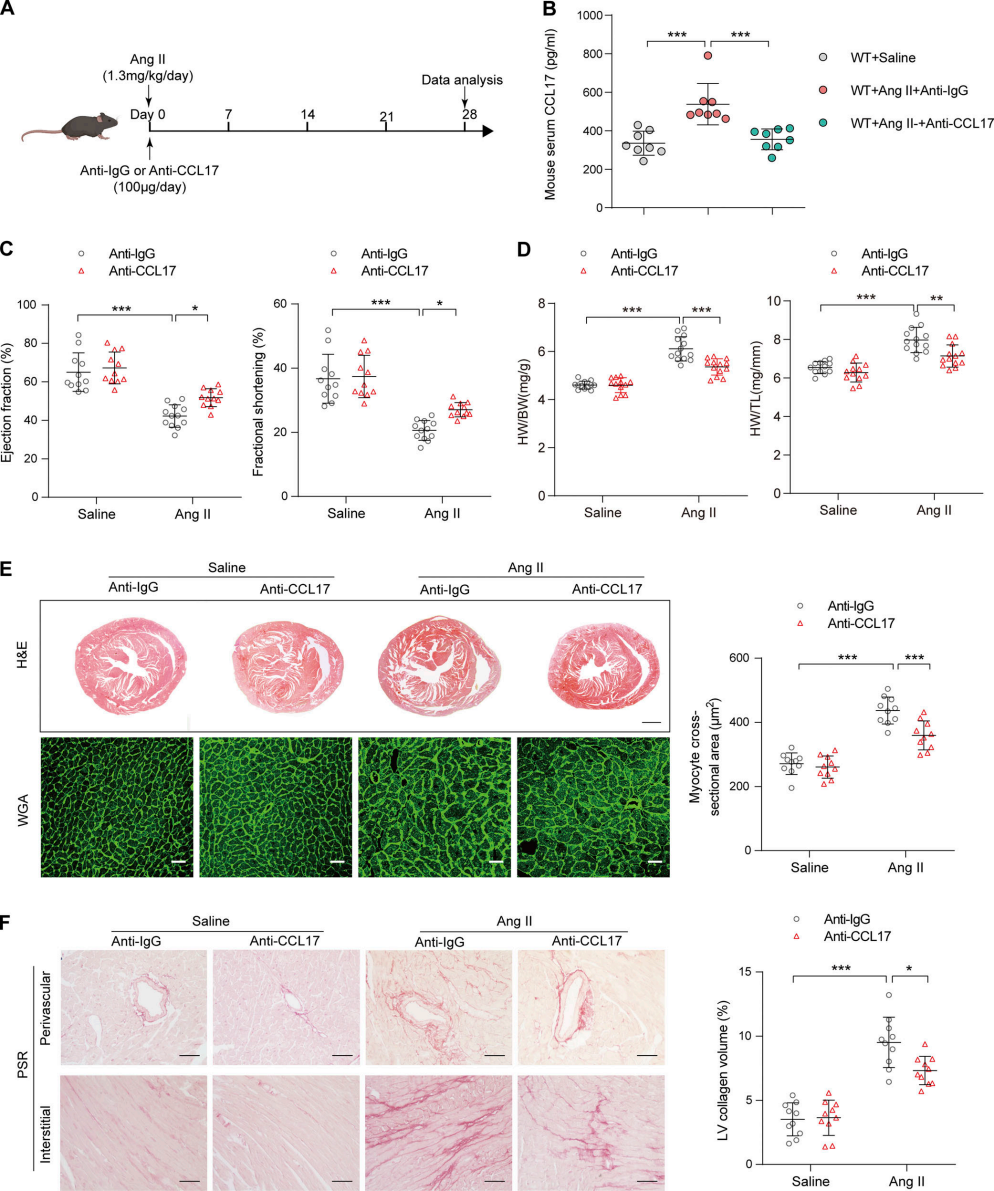
**CCL17 neutralizing antibodies attenuated Ang II–induced cardiac hypertrophy and HF. (A)** 8–12-wk-old C57BL/6J background WT mice were treated with isotype IgG or anti-CCL17 antibodies (100 μg/mouse/d) 28 d after saline or Ang II infusion. **(B)** Quantitative analysis of CCL17 levels in serum from saline- or Ang II–infused C57BL/6J background WT mice (8–12 wk old) treated with IgG control or anti-CCL17 antibodies (*n* = 8 biological replicates). **(C)** Measurement of EF and FS in saline- or Ang II–infused C57BL/6J background WT mice (8–12 wk old) treated with IgG control or anti-CCL17 antibody (*n* = 11–12 biological replicates). **(D)** HW/BW and HW/TL ratios of saline- or Ang II–infused mice (8–12 wk old) treated with IgG control or anti-CCL17 antibodies (*n* = 12–13 biological replicates). **(E)** Heart cross-sections were stained with H&E (top). Scale bars: 1 mm. WGA (bottom) staining of heart sections and corresponding group quantification data (right; *n* = 10 biological replicates). Scale bars: 30 µm. **(F)** PSR (left) staining and corresponding group quantification data (right) of saline- or Ang II–infused C57BL/6J-background WT mice (8–12 wk old) treated with IgG control or anti-CCL17 antibodies (*n* = 10 biological replicates). Scale bars: 50 µm. Data shown in (B–F) are presented as the mean ± SD. Statistical significance was tested and obtained using one-way ANOVA, followed by the Bonferroni post hoc test (B–F). *, P < 0.05; **, P < 0.01; ***, P < 0.001.

**Figure S5.**
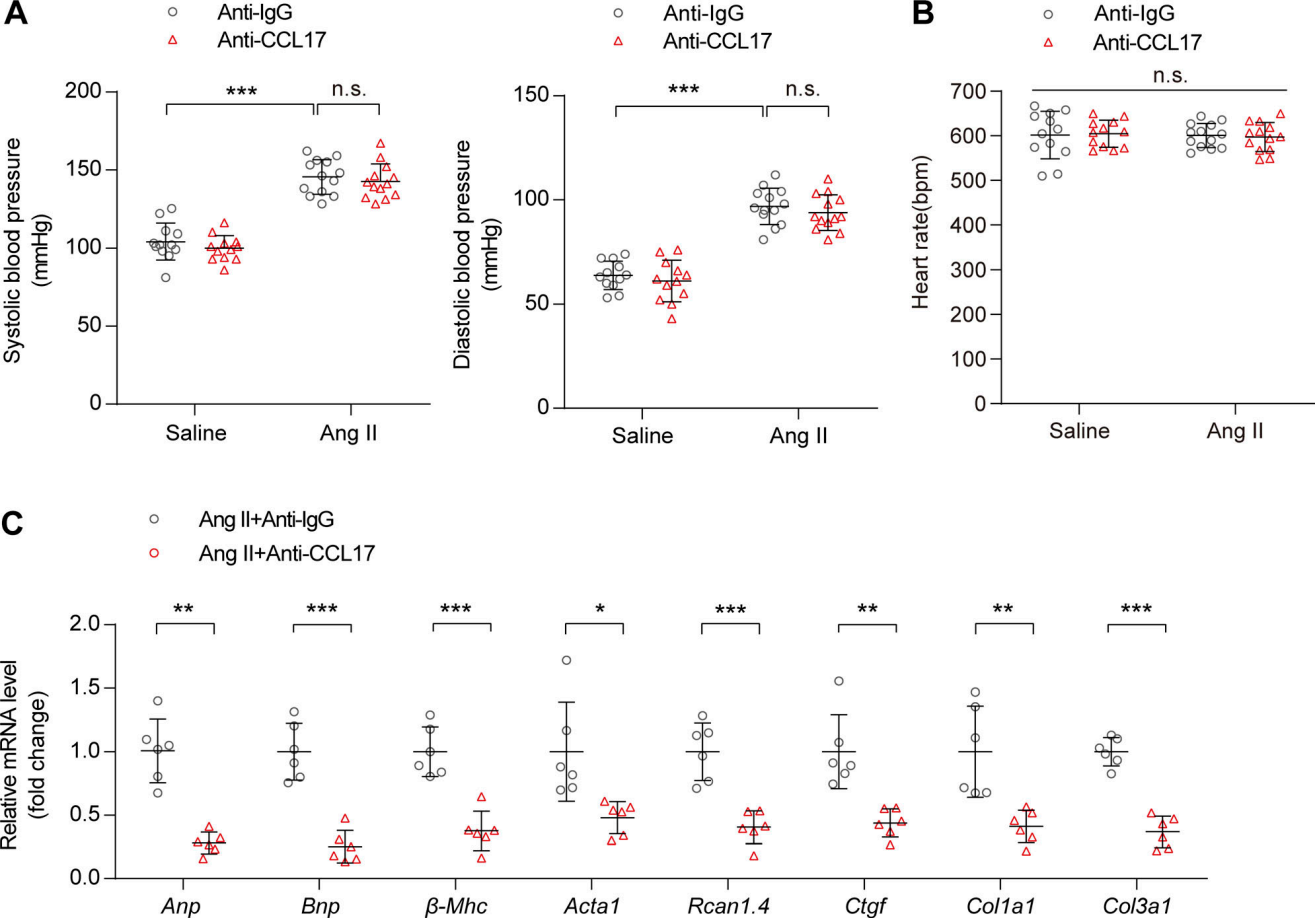
**Basic physiological indicators and cardiac functional biomarkers after the injection of CCL17 neutralizing antibody in mice.** 8–12-wk-old C57BL/6J background WT mice were treated with IgG control or anti-CCL17 antibody 28 d after saline or Ang II infusion. **(A and B)** Systolic and diastolic blood pressures (A) and heart rates (B) in C57BL/6J background WT mice treated with IgG control or anti-CCL17 antibody after saline or Ang II infusion (*n* = 12–13 biological replicates). **(C)** qRT-PCR was performed to analyze the mRNA levels of cardiac function (*Anp*, *Bnp*), hypertrophic (*β-Mhc*, *Acta1*, *Rcan1.4*), and fibrosis (*Ctgf*, *Col1a1*, *Col3a1*) marker (*n* = 6 biological replicates). Data shown in A–C are presented as the mean ± SD. Statistical significance was tested using one-way ANOVA followed by Bonferroni post hoc test (A and B). Unpaired Student’s *t*-test was used for equal variance or Welch *t*-test used for unequal variance (C). *, P < 0.05; **, P < 0.01; ***, P < 0.001.

To determine whether CCL17 inhibition ameliorates Ang II–induced pathological cardiac hypertrophy, WT mice were simultaneously treated with anti-CCL17 antibodies or isotype IgG after a 4-wk saline or Ang II infusion (Fig. 5 A). A relatively high serum CCL17 level was observed after Ang II exposure, and it was almost completely diminished by the administration of CCL17-neutralizing antibodies (Fig. 5 B) without impact on blood pressure and heart rate (Fig. S5, A and B). Compared with IgG controls, CCL17 antibody-treated mice exhibited remarkable attenuation of the Ang II–induced cardiac dysfunction, increases in the myocyte area, and myocardial fibrosis (Fig. 5, C–F). Additionally, a corresponding reduction in cardiac function, hypertrophy, and fibrosis-gene expression was noted (Fig. S5 C).

**Figure 6.**
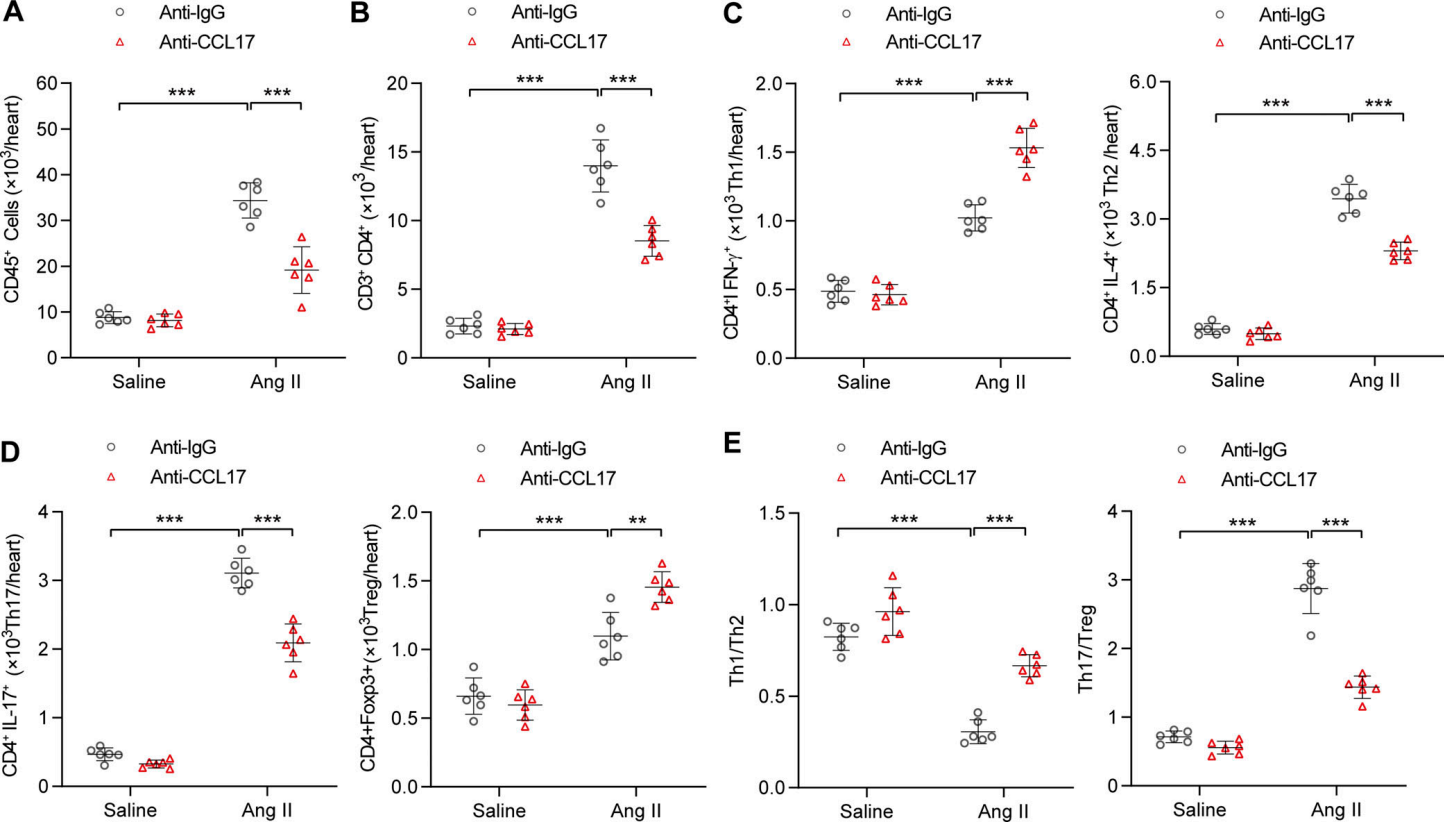
**CCL17 neutralizing antibodies inhibited Ang II–induced inflammatory response. (A–D)** Flow cytometry analysis of CD45^+^ T cells (A), CD3^+^CD4^+^ T cells (B), and T cell subsets (C and D) of saline- or Ang II–infused C57BL/6J background WT mice (8–12 wk old) treated with IgG control or anti-CCL17 antibodies (*n* = 6 biological replicates). **(E)** Th1/Th2 and Th17/Treg ratios in C57BL/6J background WT mice (8–12 wk old) treated with IgG control or anti-CCL17 antibodies after saline or Ang II infusion (*n* = 6 biological replicates). All data are presented as the mean ± SD. The Kolmogorov–Smirnov normality test was used to assess data distribution. Statistical significance was tested and obtained using one-way ANOVA, followed by the Bonferroni post hoc test (A–E). **, P < 0.01; ***, P < 0.001.

Consistent with findings from *Ccl17*-KO mice, flow cytometry revealed that the inflammatory reaction and expansion of CD45^+^ and CD3^+^CD4^+^ T cells were significantly lower in mice treated with CCL17 neutralized antibodies (Fig. 6, A and B). In terms of T cell subsets, the infiltration of Th2 and Th17 cells was reduced and the infiltration of Th1 and Treg cells was increased after CCL17 neutralized antibody treatment in Ang II–induced pathological cardiac remodeling (Fig. 6, C and D). Except for the reduction in the Th17/Treg ratio, the CCL17 antibody-treated group displayed preferential Th1-type polarization (Fig. 6 E).

## Discussion

In the present study, our findings demonstrated that circulating CCL17 plays a key role in aging as well as Ang II–induced pathological cardiac remodeling and HF. Aging or Ang II infusion significantly upregulated circulatory CCL17 levels and hypertrophic hearts. *Ccl17*-KO ameliorates age-related and Ang II–induced cardiac hypertrophy, fibrosis, and inflammatory response. In addition, the administration of anti-CCL17 neutralizing antibodies markedly attenuated Ang II–induced cardiac dysfunction. This mechanistic study revealed that CCL17 recruits Th2 cells through binding to CCR4, thus perturbing T cell subset balance, promoting fibrotic-factor release (predominantly IL-4 and IL-17), and resulting in cardiac hypertrophy, fibrosis, and subsequent HF.

As circulating proteomic signatures of age are closely associated with aging and age-related diseases, the proteomic features of aging and their genetic architecture in systems biology (Emilsson et al., 2018; Lehallier et al., 2019; Sun et al., 2018) have been progressively elucidated. However, whether circulating proteins associated with chronological age constitute a potential therapeutic target for key aging diseases, such as pathological cardiac remodeling and HF, remains unclear. Based on SOMAscan aptamer technology, we and Lehallier et al. (2019) both found the CCL17 level to be significantly associated with age. As regards our SOMAscan assay, probably due to the relatively small sample size, the P value in our study was at a critical level (allpfdr value = 0.0485, as shown in Table S2). Nevertheless, a subsequent ongoing community-based cohort study in the Chinese population further confirmed our results. Thus, the combination of different independent cohorts effectively prevents systematic error and race-based selectivity bias. In addition, we also found that CCL17 expression increased with age in mice. Thus, our data indicate that CCL17 is a strong candidate target for aging and age-related diseases.

Inflammation is a vital mediator of cardiovascular disease; however, the development of new therapeutic targets coupled with inflammatory biomarker utilization has struggled in HF over the past two decades (Mann, 2015; Williams et al., 2019). As a critical type of cytokine, chemokines orchestrate immune-cell migration and positioning in homeostasis. There is mounting evidence suggesting that chemokines play a key role in HF. For example, the chemokine CCL5 (Montecucco et al., 2012) and CXCL1–CXCR2 axis (Wang et al., 2018) have been reported to play important roles in cardiac remodeling and HF. However, the role of chemokines as a possible drug target for HF in humans has yet to be explored. Moreover, there is still no drug that targets secreted proteins with a proven therapeutic effect on age-related pathological cardiac remodeling and HF. Our previous studies demonstrated that serum CCL17 levels are associated with coronary artery disease and atherosclerosis severity, independently of traditional cardiovascular risk factors (Ye et al., 2017; Ye et al., 2015; Ye et al., 2014). In the present study, we demonstrated that CCL17 deficiency and the therapeutic administration of CCL17 neutralizing antibodies attenuated Ang II–induced and age-related cardiac hypertrophy, fibrosis, and subsequent HF. Interestingly, we found that CCL17 was difficult to detect in normal myocardial tissue; nonetheless, it exhibited an explosive increase due to the inflammatory cascade in pathological cardiac hypertrophy. CCL17 has been reported to be constitutively expressed in the thymus and produced by DCs, endothelial cells, keratinocytes, and bronchial epithelial cells (Bajpai et al., 2018; Saeki and Tamaki, 2006; Weber et al., 2011). CCL17 upregulation may be predominantly attributed to two pathways in the present study. One of the most important pathways derives from CCL17-expressing DCs belonging to CD11b^+^CD8^−^Dec205^+^ DC subsets in peripheral nonlymphoid organs, which is consistent with the prolific infiltration of DCs in the failing heart (Alferink et al., 2003; Ismahil et al., 2014; Laroumanie et al., 2014). Another possible source of CCL17 in heart tissue is the circulatory system, which abounds with secreted cytokines produced by immune tissues and immune cells. CCL17 deficiency has been reported to potentially prolong cardiac graft survival (Alferink et al., 2003) and improve the symptoms of central nervous system diseases (Fulle et al., 2018; Scheu et al., 2017). In this sense, circulating CCL17 blockade can improve the overall health status, similar to what has been observed in the aged *Ccl17*-KO mice.

The expansion and activation of T cells in chronic HF are indispensable in the cardiac remodeling process (Bansal et al., 2017; Martini et al., 2019; Ngwenyama et al., 2021). Additionally, the relative plasticity and differentiation of CD4^+^ T cell subsets are a key factor in the development of immune-mediated diseases, and specific cytokine profiles secreted by different CD4^+^ T cell subsets (including Th1 cells that produce IFN-γ and IL-2; Th2 cells that secrete IL-4, IL-5, and IL-13; Th17 cells that produce IL-17; and Tregs) constitute a crucial mechanism in the defense against pathogens (Geginat et al., 2014). Our cytokines array data verified that CCL17 knockout mainly affects the secretion of Th1 and Th2 cytokines, but not other cytokines, and we need to carry out further experiments to verify this finding and the underlying mechanisms subsequently.

The chemokine CCL17 has been proven to preferentially promote T cell responses with Th2 polarization via CCR4; however, controversy exists regarding whether CCL17 potentially participates in the recruitment of Tregs (Feng et al., 2022; Iellem et al., 2001; Lee et al., 2005). Recently, Feng et al., (2022) have reported that CCL17 inhibited Treg recruitment through biased activation of CCR4 in myocardial injury. Our findings indicate that the predominance of Th2 and Th17 (compared with that of Th1 and Treg, respectively) in failing hearts was interrupted by CCL17 deficiency or neutralization. The changes in the Th1/Th2 cell ratio can be directly attributed to the activation of CCR4 on Th2 cells, while the decrease in the Th17/Treg cell ratio after blocking CCL17 was likely regulated by the inhibitory effect of CCL17 on Treg cells. Moreover, our study strongly supports previous conclusions suggesting that the Th1-to-Th2 shift is important in senescence-associated fibrosis (Biernacka and Frangogiannis, 2011). In addition, Belperio et al. (2004) reported that CCL17 neutralization, but not that of CCL22, led to a reduction in Th2-mediated pulmonary fibrosis. CCL17 has also been shown to be involved in Th2-mediated cystic fibrosis (Tiringer et al., 2013), peritoneal fibrosis (Terri et al., 2021), and the granuloma formation of sarcoidosis (Nguyen et al., 2018). Moreover, CCL17 has been confirmed as a circulating biomarker of fibrosis-associated diseases, such as idiopathic pulmonary fibrosis (Sivakumar et al., 2021), cystic fibrosis (Adib-Conquy et al., 2008), peritoneal (Chen et al., 2020), and renal fibrosis (Hsieh et al., 2021; Pai et al., 2020). Our present study is the first to link CCL17 directly to age-related and Ang II–induced cardiac fibrosis. Collectively, these findings strongly suggest the critical roles of CCL17 and the Th2-mediated immune response in fibrosis across organs. Therefore, CCL17 blockade may exert its cardioprotective effect by interrupting inflammatory-cascade amplification, thus further resulting in the alleviation of cardiac hypertrophy and fibrosis.

The current study had certain limitations. To date, myocardial interstitial fibrosis has been considered an important pathological feature of cardiac remodeling that determines the progression of HF. As fibrosis level increases, cardiac function gradually decreases. It has been verified in both clinical cohort (Rosello-Lleti et al., 2007) and animal model (Frangogiannis, 2021; Peng et al., 2015) studies that the Th2 cytokine IL-4 participates in HF progression by promoting myocardial fibrosis. In the future, further evidence to confirm the necessity of IL-4 for the involvement of CCL17 in pathological cardiac remodeling is warranted. Second, compared with cross-sectional and correlation studies, a prospective cohort study investigating the relationship between CCL17 and HF could provide more convincing evidence for therapeutic intervention. To the best of our knowledge, this study is the first to reveal CC17 as a novel therapeutic target in age-related and Ang II–induced pathological cardiac dysfunction. Although we found CCL17 deficiency to contribute to the overall health status in aged mice, the role of CCL17 as aging-controlling protein warrants further investigation.

In conclusion, population studies and experiments based on the disease model of age-related and Ang II–induced pathological cardiac hypertrophy have together revealed the destructive effect of CCL17 on cardiac tissue. Elevated serum CCL17 levels were associated with cardiac function deterioration, suggesting that CCL17 may be a new biomarker of HF. Furthermore, the administration of CCL17-blocking agents may be a novel anti-inflammatory strategy for HF.

## Materials and methods

### Study population and design

The 240 healthy men and women (aged 22–93 yr) in our study were from the Baltimore Longitudinal Study of Aging and Genetic and Epigenetic Signatures of Translational Aging Laboratory Testing studies, and their detailed characteristics were described in Table S1 (Tanaka et al., 2018). Subjects were categorized into 15-yr age strata (20–35, 35–50, 50–65, 65–80, and 80+ yr) and analyzed for differences between age groups (48 subjects per strata; Table S2). To reduce the underlying bias associated with disease and explore age-related differences independent of disease, we selected individuals meeting the strict health criteria originally described in the Baltimore Longitudinal Study of Aging (Shock et al., 1984). The study protocol was approved by the Internal Review Board of the Intramural Research Program of the National Institutes of Health, and all participants provided written informed consent.

The community-based Shunyi cohort study in China was designed to explore the risk factors for cardio-cerebro-vascular and age-related diseases. Between June 2013 and April 2016, a total of 1,041 inhabitants of three natural villages in Shunyi, Beijing, were enrolled. The clinical data of all participants, including standardized questionnaires, physical examination, and biochemical testing, were collected. Moreover, all subjects were invited to undergo serum CCL17 level measurement. Although 230 participants refused to participate in our study (response rate, 77.9%), we found no differences in baseline characteristics between the CCL17 testing population and the total population (Table S3). Furthermore, we performed a PCA of 13 clinical indexes (including CCL17 and BMI obtained from height and weight; thus, they were not repeated) in all patients, using the princomp function in R and default parameters. The PCA results for individual patients were generated using the fviz_pca_ind function in R. We also found that the CCL17 testing population was matched to the total population (Fig. S1 B).

To validate the correlation between circulating CCL17 and cardiac function, 32 hospitalized patients with chronic HF and 32 normal controls matched by age and sex without any underlying diseases were enrolled. In addition, another clinical cohort of 17 patients with acute decompensated HF was recruited from a single center (with a history of chronic HF). Patients with HF were diagnosed according to the “2021 ESC Guidelines for the diagnosis and treatment of acute and chronic heart failure” (McDonagh et al., 2021). The baseline characteristics of these two cohort studies are shown in Tables S4 and S5.

The LV transcriptomes were downloaded from the GEO database (GSE141910), which contained 366 samples, including 200 HF and 166 non-HF samples. All data were processed as previously described (Xing et al., 2021).

Informed consent was obtained from each subject from the community-based Shunyi cohort study or HF study. All investigations were approved by the Ethics Committee of Peking Union Medical College Hospital and conducted in accordance with the principles of the Declaration of Helsinki (reference number: B-160 and HS-2031).

### Proteomic assessment and bioinformatics analysis

To characterize the proteomic signature of chronological age, proteomic profiling was conducted on 240 healthy men and women aged 22–93 yr. Thereafter, we classified subjects into five categories and age strata: 20–35, 35–50, 50–65, 65–80, and 80+ yr, corresponding to age groups 1–5, respectively.

A total of 1,301 proteins were measured in plasma using the SOMAscan assay. The association of each protein with chronological age was assessed using a linear regression model adjusted for sex, site, batch, and race (white, black, or other). We defined subjects aged >65 yr as old people and ran a linear model comparing each of the age groups (65–80, 80+) with the reference group (20–35). β1-4/se1-4/p1-4 represented the comparisons of age groups 2–5 with age group 1, individually. The overall significance of age on protein levels (p1–4) was defined as allp (overall P value). Adjusted overall P values were defined as allpfdr (overall false discovery rate, Q values). The differential expression of proteins between young and older people had to satisfy three standards simultaneously: p3 < 0.05 (age 20–35 versus age 65–80), p4 < 0.05 (age 20–35 versus age >80), and allpfdr < 0.05. A total of 312 proteins fulfilled the requirements, and the results list was exported to DAVID (https://david.ncifcrf.gov/) software for functional annotation analysis (Fig. S1 A).

The proteomic study was also described in our previous study (Tanaka et al., 2018). Proteomic data generated from this study are available upon request (tanakato@mail.nih.gov). Investigators had access to all raw data upon request and National Institutes of Health authorization.

### Animals

All animal experiments were approved by the Animal Care and Use Committee of the Institute of Peking Union Medical College Hospital, Chinese Academy of Medical Sciences, and Peking Union Medical College. All male mice (C57BL/6J background) were maintained under specific pathogen–free conditions on a 12-h light/dark cycle and supplied a normal diet.

### Generation of *Ccl17*-KO mice

Global germ-line *Ccl17*-KO mice (C57BL/6J background) were generated using the CRISPR/Cas9 system in our laboratory. Initially, the sequencing primers of the target site were designed, and their PCR products (C57BL/6J mouse) were sequenced and confirmed to be equivalent to those from the National Center for Biotechnology Information and Ensembl database. As shown in Table S6, we designed a set of primers near the 5′ end of exon 1 and 3′ end of exon 3. Meanwhile, the PCR-product assay confirmed their specificity. Second, seven pairs of sgRNA primers were designed following the Addgene protocol (http://www.addgene.org/crispr/church/). The primer information of all sgRNA molecules and their software scores are listed in Table S7. Subsequently, oligos were cloned into the puc57-gRNA vector (#51132; Addgene), and the sequences were further confirmed by DNA sequencing (Table S8). sgRNA 3 and 8 were used for the subsequent step after detection by the Universal CRISPR Activity Assay, an sgRNA-activity detection system developed by Biocytogen. Thereafter, the linearized pcS7-Cas9 plasmids (Viewsolid Biotech) and puc57-sgRNA vector were extracted with phenol-chloroform and subsequently used as templates for in vitro transcription using the mMESSAGE mMACHINE T7 Kit (#AM1344; Invitrogen). The RNA products were precipitated using LiCl and purified using the Qiagen RNeasy Cleanup Kit. Superovulated female C57BL/6J mice (4–5 wk old) were mated with male mice of similar background, and fertilized embryos were collected from their oviducts. The injected zygotes were cultured in KSOM with amino acids at 37°C for ∼0.5–1 h and subsequently transferred to the uterus of a pseudopregnant female ICR mouse. Genotyping was performed from tail DNA by specific primers (forward, 5′-AAG​CAC​ACA​TGC​AGA​TAC​ACC​GTG​A-3′; reverse, 3′-CAG​GAA​GGC​AGA​CAC​ACT​CAG​AAG​G-5′) using gel electrophoresis and confirmed by sequencing. In total, we obtained nine founder mice; however, four of them died after birth. Based on gel electrophoresis results, mice #6 and #7 were used as founder mice for subsequent experiments. The genome of mouse #8 was unstable, and we speculated that it might have been off-target. Through sequencing, we found that 1,704 bases were deleted in mouse #6, while 1,707 bases were deleted in mouse #7 (Fig. S1 E). The founder mice were mated with C57BL/6J background mice to acquire the F1 generation.

### Animal models

The male *Ccl17*-KO mice and their control WT littermates were maintained for 21 mo. In addition, their corresponding young littermates were maintained for 4 mo. *Ccl17*-KO male mice and their corresponding WT littermates of 8–12 wk of age were used to establish cardiac hypertrophy by chronic subcutaneous infusion of Ang II (#A9525; Sigma-Aldrich) at a dose of 1.3 mg/kg/d for 4 wk using Alzet 2004 osmotic pumps. For treatment with neutralizing antibodies, the anti-CCL17 antibody (#MAB529; R&D Systems) and corresponding isotype control (rat IgG2a) customized in R&D were treated intraperitoneally at a dose of 100 μg/100 μl/mouse/d.

### Echocardiography and blood pressure

Mouse echocardiography was performed using a VisualSonics Vevo770 ultrasound biomicroscope (VisualSonics, Inc.) with a 15-MHz linear array ultrasound transducer. Initially, mice were anesthetized with 1.0% isoflurane, and heart rate was maintained at 400–500 beats/min. Parasternal long-axis and short-axis images acquired in B-mode were used to evaluate LV function. Thereafter, the M-mode cursor was positioned at the phase of the smallest or largest LV (end-systole or end-diastole) area. LV end-systolic diameter (LVESD) and LV end-diastolic diameter (LVEDD) were measured to indicate wall thickness and chamber dimensions. FS of LV was calculated as follows: (LVEDD − LVESD)/LVEDD. Heart rate and blood pressure were measured using a noninvasive CODA blood pressure monitoring system (Kent Scientific).

### Histopathology analysis

Heart tissues were arrested using a 10% potassium chloride solution and subsequently fixed in 4% paraformaldehyde. Thereafter, fixed hearts were dehydrated, embedded in paraffin, and subsequently cut transversely into 5-μm sections for H&E or WGA staining (#W11261; Invitrogen) to measure myocyte cross-sectional areas. The fibrosis level was detected using a PSR Staining Kit (#ab150681; Abcam). In addition, the slides were incubated with anti-CD4 (#14-0041-85; eBioscience) for T cells, anti-Ly6G (#14-5931-82; eBioscience) for neutrophils, anti-CD11c (#ab1211; Abcam) for DCs, and anti-F4/80 (#ab6640; Abcam) for macrophages. H&E and PSR staining were analyzed using the Leica Microsystem. Immunofluorescence was analyzed using laser scanning confocal microscopy (Olympus Corporation). The Image-Pro Plus 6.0 system was used for quantitative analysis.

### Rotarod test

The rotarod test was used to evaluate motor function. A 30 × 60-mm accelerating rotarod was used to evaluate motor coordination and balance (#YLS-4D; Jinan Yiyan Technology Company). The speed of the rotarod accelerated from 5 to 40 rpm over a 5-min period. Latency to fall off the rotarod was recorded with a 5-min cutoff time for three trials per day over 2 consecutive days.

### Open-field test

The open-field test was used to evaluate locomotor activity and emotional response (Ackert-Bicknell et al., 2015). This test was conducted in a quiet environment. The apparatus comprised a transparent square cage (50 × 50 × 50 cm). Each mouse was placed in the open-field apparatus and recorded for 5 min. Concurrently, the camera and timing were initiated. On entering the subsequent group, 75% alcohol was used to clean the box completely to wipe off the mice’s residual urine, smell, and hair. The total distance, average velocity, and time spent in the center area were measured. ANY-maze software was used for further analysis.

### Spontaneous alternation test

The spontaneous alternation test was conducted to assess spatial working memory (Prieur and Jadavji, 2019). The Y-maze is a three-arm maze with equal angles between each arm (30 cm in length × 8 cm in width × 30 cm in height). We encoded the arms separately, and each mouse was initially placed in the “starting” arm of the maze. The number and sequence of subsequent arm entries were recorded on camera over 10 min. On entering the subsequent group, 75% alcohol was used to clean the box completely to wipe out the mice’s residual urine, smell, and hair. ANY-maze software was used for further analysis. Alternation score (%) = ([number of alternations]/[total arm entries − 2]) × 100%.

### Cardiac immune-cell isolation

The protocol for cardiac immune-cell isolation was provided by Dr. Sumanth D. Prabhu (Bansal et al., 2017). Beforehand, the heart tissues from sacrificed mice were dissected and subsequently immersed in cold PBS in a cell culture dish. Thereafter, fat, blood clots, and connective tissue were removed using fine scissors and forceps. Second, a razor blade was used to mince the heart in a 1.5-ml EP tube, and the minced heart was subsequently transferred to a 50-ml tube. Thereafter, 5-ml PBS solution was added (pellet was dissociated by squirting the PBS and by hand) and the tubes were placed in ice. After digestion with 0.4% collagenase I, II, IV (#C0130, #C5138, #C6885; Sigma-Aldrich), and 0.25% trypsin (#25200056; Gibco) in PBS for 5 min (six times), the digested heart-tissue suspensions were filtered through a 50-μm cell strainer. Finally, the filtrate was centrifuged at 500 *g* for 8 min and the supernatant was discarded. MACS buffer (150 μl) was added, and fixed cells were resuspended and stored for further flow cytometry analysis.

### Flow cytometry and antibody assay

Eight-color flow cytometric data were acquired using an Attune Nxt flow cytometer (Thermo Fisher Scientific). This eight-color panel comprised antimouse FVD (FVD eFluor 506, #65-0866; eBioscience), CD45 (BV605, #563053; BD Bioscience), CD3 (AF700, #557984; BD Bioscience), CD4 (BV786, #563331; BD Bioscience), IFN-γ (PE-CF594, #562303; BD Bioscience), IL-4 (PE, #554435; BD Bioscience), IL-17A (BV421, #563354; BD Bioscience), and Foxp3 (AF647, #563486; BD Bioscience) fluorochrome-conjugated antibodies. These markers represent a standard immunophenotypic panel for determining the percentages of white (CD45^+^), CD4 T (CD3^+^CD4^+^), Th1 (CD4^+^IFN-γ^+^), Th2 (CD4^+^IL-4^+^), Th17 (CD4^+^IL-17^+^), and Treg (CD4^+^ Foxp3^+^) cells.

Heart tissues were digested into single-cell suspensions. Before addition to FACS tubes (#352054; BD Bioscience), samples were treated with OptiLyse (#555899; BD Bioscience) for 15 min in the ice, and living status was detected by FVD. Individual cells were washed twice with PBS, subsequently re-suspended in staining buffer, and incubated with monoclonal antibodies targeting surface antigens CD45, CD3, and CD4 at room temperature for 15 min in the dark. After washing with buffer (10% bovine serum albumin in PBS), the cells were fixed with a fixation/permeabilization kit (#562574; BD Bioscience) for 45 min, according to the manufacturer’s protocol. Furthermore, cells were washed twice in wash buffer and subsequently incubated with antibodies targeting intracellular cytokines IFN-γ, IL-4, IL-17A, and the transcription factor Foxp3 with Brilliant Stain Buffer (#563794; BD Bioscience) for 40 min. Finally, after washing with buffer twice, all samples were acquired using the Attune Nxt flow cytometer (Thermo Fisher Scientific) and analyzed using FCS EXPRESS software (De Novo Software).

### qRT-PCR

Total RNA was extracted using the Qiagen RNeasy Mini Kit (#74104; Qiagen), and first-stand cDNA was synthesized using the PrimeScript RT Master Mix (#RR036Q; Takara). qRT-PCR with AceQ qPCR SYBR Green Master Mix (Q121-02; Vazyme) was performed to examine the relative mRNA levels of indicated genes. Sequences for qRT-PCR primers are shown in Table S9.

### Human blood sample collection

All human blood sample collections were performed with the approval of the Ethics Committee of Peking Union Medical College Hospital and followed the principles outlined in the Declaration of Helsinki. All study patients and subjects signed an informed consent form. The plasma samples of patients with HF were collected from the Department of Cardiology, Peking Union Medical College Hospital. According to the criteria set out in the guidelines, the diagnosis of HF in all patients was confirmed by at least two senior doctors. Patients with acute decompensated HF were diagnosed based on the presence of at least one symptom (dyspnea, orthopnea, or edema) and one sign (rales, peripheral edema, ascites, or pulmonary vascular congestion on chest radiography) of HF. Additionally, another eligibility criterion was the history of chronic HF. The first plasma sample from a patient with acute decompensated HF was collected within the preceding 24 h, and the second was acquired at discharge.

### Cytokine measurements

CCL17 serum levels in humans and mice were measured using a human or mouse CCL17/TARC ELISA kit, respectively (#PDDN00, #MCC170; R&D Systems), according to the manufacturer’s instructions. CCL22 serum levels in mice were measured using a mouse CCL22/MDC ELISA kit, according to the manufacturer’s instructions (#MCC220; R&D Systems). Standard cytokine dilutions were initially prepared, with subsequent additions of 50 μl of standard, control, or sample per well. Thereafter, each well was aspirated and washed, repeating the process four times and adding 100 μl of mouse or human TARC conjugate to each well. Finally, 100 μl of substrate solution and 100 μl of stop solution were added to each well. The signals were detected using a Labsystems Multiskan mass spectrometry spectrophotometer (Thermo Labsystems) and calculated using Ascent software v2.6 (Thermo Labsystems).

### Western blotting

Protein was extracted from frozen heart tissues using radioimmunoprecipitation assay buffer (#P0013B; Beyotime), which contains protease/phosphatase inhibitors. After sonication and centrifugation, the supernatants were collected to quantify concentration using a BCA Protein Assay Kit (#23235; Thermo Fisher Scientific) before Western blotting. Suitable protein was separated on SDS-polyacrylamide electrophoresis gels and subsequently transferred to polyvinylidene difluoride membranes (Millipore). Thereafter, 5% no-fat milk was used to incubate the membranes. The membranes were washed in Tris-buffered saline with tween thrice at room temperature and subsequently incubated with the following primary antibodies at 4°C overnight: anti-TARC (#ab182793; Abcam) and anti-GAPDH (#ab8245; Abcam). After washing with Tris-buffered saline with tween thrice, all membranes were incubated with an HRP-conjugated secondary antibody (#ZB2301; ZSGB-BIO) and exposed to Pierce ECL Western Blotting Substrate (#32106; Thermo Fisher Scientific) for the measurement of protein expression.

### Protein microarray

To detect the cytokines produced by T cell subsets, a Mouse Th1/Th2/Th17 Cytokines Array (#QAM-Th17-1; Ray Biotech, Inc.) was used, according to the manufacturer’s instruction. Initially, standard cytokine dilutions were prepared. Thereafter, 100-μl sample diluents were added to each well and incubated at room temperature for 30 min on block slides. Subsequently, 100-μl standard cytokines or samples were added to each well and incubated in arrays at room temperature for 1–2 h. After washing five times (5 min each) with a 150-μl 1× wash buffer, samples were subsequently incubated with a biotinylated antibody cocktail and Cy3-equivalent dye-conjugated streptavidin. Finally, the samples were decanted from each well and washed five times with wash buffer at room temperature with gentle rocking to completely remove the wash buffer at each wash step. Signals were detected by an InnoScan 300 Microarray Scanner, and the primary data are shown in Table S10.

### Statistical analysis

All data are presented as the mean ± SD in triplicates. Unless otherwise stated, differences were considered statistically significant at P < 0.05. The Shapiro–Wilk or Kolmogorov–Smirnov test was used to assess data normality. Log transformation was used for non-normally distributed data before the analysis of variance. Moreover, variance homogeneity was assessed using the F-test (two groups) or Brown–Forsythe test (three or more groups). Thereafter, the unpaired or paired standard Student’s *t*-test was used for equal variance or the Welch *t*-test for unequal variance (unpaired standard Student’s *t*-test). Where there were more than three groups, P values were obtained using one-way ANOVA, followed by the Bonferroni post hoc test, provided the assumptions (equal variance and normal distribution) were satisfied. We used the nonparametric Kruskal–Wallis test, followed by Dunn’s post hoc test, to correct for multiple comparisons. In addition, categorical variables are presented as counts and percentages, and they were compared using the χ^2^ test.

For healthy subjects, a basic model of linear regression adjusted for sex, site, batch, and race was used to assess the association of each protein with chronological age. In addition, linear regression analysis, adjusted for sex, BMI, hypertension, smoking status, LDL-C, and diabetes mellitus, was used to explore the associations between age and serum CCL17 concentrations. All analyses were performed using GraphPad Prism 9.0 or SPSS Version 21 software.

### Online Supplemental Material

Fig. S1 shows chemokine CCL17 exhibited an age-dependent increase and generation and validation of the *Ccl17*-KO mouse model. Fig S2 shows age-related changes in grip and behavior. Fig. S3 contains basic characteristics of WT and *Ccl17*-KO mice in age-related and Ang II–induced models. Fig. S4 shows accumulation of CD45^+^ cells, CD4^+^ T cells, macrophages, DCs, and neutrophils within the cardiac tissue of WT mice after infusion with Ang II for 4 wk. Fig. S5 shows basic physiological indicators and cardiac functional biomarkers after the injection of CCL17 neutralizing antibody in mice. Table S1 shows primary data of 1,301 proteins after adjusted linear regression. Table S2 shows age-related differential expression proteins. Table S3 shows baseline characteristics of the study population from the Shunyi cohor. Table S4 shows baseline characteristics of the patients with chronic HF and healthy controls. Table S5 shows baseline characteristics of the patients with acute decompensated HF. Table S6 lists sequencing primer design of the target site. Table S7 lists primer information and software scores of sgRNA. Table S8 shows sgRNA oligos design. Table S9 shows the primer list for qRT-PCR. Table S10 shows primary data of the protein microarray.

## Supplementary Material

Table S1 shows primary data of 1,301 proteins after adjusted linear regression.

Table S2 shows age-related differential expression proteins.

Table S3 shows baseline characteristics of the study population from the Shunyi cohort.

Table S4 shows baseline characteristics of the patients with chronic HF and healthy controls.

Table S5 shows baseline characteristics of the patients with acute decompensated HF.

Table S6 lists sequencing primer design of the target site.

Table S7 lists primer information and software scores of sgRNA.

Table S8 shows sgRNA oligos design.

Table S9 shows the primer list for qRT-PCR.

Table S10 shows primary data of the protein microarray.
